# Early-Pregnancy Metabolic Syndrome and Subsequent Incidence in Gestational Diabetes Mellitus in Arab Women

**DOI:** 10.3389/fendo.2020.00098

**Published:** 2020-02-27

**Authors:** Kaiser Wani, Shaun Sabico, Abdullah M. Alnaami, Sara Al-Musharaf, Mona A. Fouda, Iqbal Z. Turkestani, Abdulrahman Al-Ajlan, Naemah M. Alshingetti, Majed S. Alokail, Nasser M. Al-Daghri

**Affiliations:** ^1^Chair for Biomarkers of Chronic Diseases, Department of Biochemistry, College of Science, King Saud University, Riyadh, Saudi Arabia; ^2^Department of Community Health, College of Applied Medical Science, King Saud University, Riyadh, Saudi Arabia; ^3^Endocrinology Division, Department of Medicine, College of Medicine, King Saud University, Riyadh, Saudi Arabia; ^4^Department of Obstetrics and Gynaecology, College of Medicine, King Saud University, Riyadh, Saudi Arabia; ^5^Department of Clinical Lab Sciences, College of Applied Medical Sciences, King Saud University, Riyadh, Saudi Arabia; ^6^Department of Obstetrics and Gynaecology, King Salman Bin Abdulaziz Hospital, Riyadh, Saudi Arabia

**Keywords:** gestational diabetes mellitus, OGTT, insulin resistance, metabolic syndrome, hypertriglyceridemia, hyperglycemia

## Abstract

**Introduction:** This study aimed to investigate the association between components of metabolic syndrome (MetS) at first trimester and development of Gestational diabetes mellitus (GDM) in 498 Saudi pregnant women.

**Materials and Methods:** Biochemical and anthropometric parameters were determined at the first trimester and MetS components were defined. Participants were screened for GDM at follow up according to International Association of Diabetes and Pregnancy Study Group (IADPSG) criteria. The main outcome measures were development of GDM and GDM risk vs. MetS components at first trimester.

**Results:** One hundred twenty three (24.7%) were diagnosed with GDM according to IADPSG criteria. GDM risk was significantly higher for participants with hypertriglyceridemia at 1st trimester even after adjusting for age, BMI and parity (OR: 1.82; CI: 1.1–3.7, *p* = 0.04). Furthermore, the odds of hyperglycemia at 1st trimester was significantly higher in GDM than in non-GDM participants even after adjustments (OR: 2.13, 95% CI: 1.1 to 4.3, *p* = 0.038). The receiver operating characteristic (ROC) for predicting GDM revealed an area under the curve (AUC) of 0.69 (95% CI: 0.64 to 0.74, *p* < 0.001) and 0.71 (95% CI: 0.65 to 0.77, *p* < 0.001) for first-trimester hyperglycemia and hypertriglyceridemia respectively.

**Conclusions:** The incidence of GDM in Saudi pregnant women was strongly associated with hyperglycemia and hypertriglyceridemia at first trimester. These findings are of clinical importance, as an assessment of MetS in early pregnancy can identify women at higher risk of developing GDM.

## Introduction

The prevalence of gestational diabetes mellitus (GDM) is increasing globally (1, 2). GDM, defined as an impairment in glucose regulation during pregnancy, has serious short term and long term health outcomes for both mother and child including preterm delivery; cesarean delivery; excessive fetal growth; neonatal hyperinsulinemia, hypoglycemia, and hyperbilirubinemia etc. (3–5). Furthermore, although glucose regulation normalizes immediately after delivery, studies suggest that women who had GDM are at an increased risk of progressing to type 2 diabetes mellitus (T2DM) and their infants are at a higher risk for childhood obesity (5, 6).

GDM usually appears in the latter half of pregnancy. Hence, GDM screening, according to the recommendations given by the International Association of Diabetes and Pregnancy Study Group (IADPSG) is done anytime between the latter half to the end of the second trimester. The rationale behind this is that the impairment in glucose regulation found in GDM is linked to the placental hormone-mediated insulin resistance which increases as pregnancy advances (7, 8). GDM screening, however, allows only a brief window of implementing interventions such as special diet, regular monitoring, insulin or oral agents to improve blood sugar levels in case of detection of GDM. For earlier intervention, researchers are looking at the possibility of identifying women at risk for GDM, in the early pregnancy particularly in the first trimester (9).

In recent studies, abnormal levels of various biomarkers for insulin resistance and inflammation during the first trimester like lower sex hormone binding globulin (SHBG) (10); increased placental growth factor (11); elevated C-reactive protein (12); and in our recent study, vitamin D deficiency (13); were found to be associated with GDM. Metabolic syndrome (MetS), according to many meta-analyses and clinical studies recently done, is shown to be highly predictive of new-onset type 2 diabetes (T2DM) in many different populations (14, 15). Identical to T2DM, GDM also results because of insulin resistance and impaired pancreatic insulin secretion (16). Whether MetS or its individual components are similarly associated with the development of GDM is relatively unknown, more so in a homogenous Arab population. The present study, therefore aimed to investigate the association between various components of MetS in the early pregnancy with the status of GDM done in later half of pregnancy. We hypothesize that the status of MetS and its components, measured early in pregnancy, are associated with the subsequent development of GDM.

## Materials and Methods

This longitudinal study was approved by the Ethics Committee of the College of Science, King Saud University; Riyadh, Saudi Arabia (Approval#14/4067/IRB, dated: 11.02.2014). All procedures followed were in accordance with the ethical standards of the responsible committee on human experimentation (institutional and national) and with the Helsinki Declaration of 1975, as revised in 2008. Written informed consent was obtained from each participant prior to inclusion of this study.

The study participants were recruited from three hospitals around Riyadh: King Fahad Medical City (KFMC), King Khalid University Hospital (KKUH) and King Salman Hospital (KSH). The inclusion criteria was normal pregnant Saudi women, aged 18–35 years, in their early pregnancy (gestational age <15 weeks) and carrying singleton pregnancy. Pregnant women with known multiple pregnancy; previous history of GDM or those with a history of chronic diseases like T2DM, renal or liver diseases etc; were excluded. A total of 498 Saudi pregnant women (age 29.0 ± 5.5 years) were found eligible. Optimal sample size required was 438 when power of analysis was set at 80%, confidence level set at 5%, and the frequency of outcome set at 10%. A written informed consent was taken from each participant prior to inclusion.

All participants were invited for a fasting blood withdrawal procedure and anthropometric measurements as previously described (17). In brief, the anthropometrics included height (cm), weight (kg), waist, and hip circumferences (cm), systolic and diastolic blood pressure (mmHg) which was measured using standard routine procedures. The fasting blood samples taken at this visit were immediately transported to the Chair for Biomarkers in Chronic Diseases (CBCD) in King Saud University where they were processed, aliquoted and stored at recommended temperature for further analysis.

Fasting blood samples collected at the first visit were analyzed for different biochemical estimations. Glucose, lipid profile, and calcium were quantified using routine biochemical tests in an automated biochemistry analyzer (Konelab 20, Thermo-Fischer Scientific, Espoo, Finland). The reagents were purchased from Thermo Fischer (catalogue# 981379 for glucose; 981812 for total cholesterol; 981823 for HDL-cholesterol, 981301 for triglyceride, 981367 for calcium and 981359 for alkaline phosphatase). The imprecision calculated as the total CV was ≤5%, ≤3.5%, ≤4%, ≤4%, ≤4.5%, and ≤4% for these tests respectively. Serum 25(OH) vitamin D was quantified using COBAS e-411 autoanalayzer (Roche Diagnostics, Mannheim, Germany) with commercially available immunodiagnostic system kits (IDS Ltd, Boldon Colliery, UK, Reference# 05894913190). The standards and controls used for these biochemical assays were routinely tested by the Quality assurance department of KSU, for highly reproducible research data.

Participants were invited for a follow up visit to the hospital in the later stages of pregnancy (age of gestation 27.1 ± 4.1 weeks) for routine GDM screening. The criteria used was according to the guidelines set by International Association for Diabetes in Pregnancy Society Group (IADPSG) (18). Briefly, an oral glucose tolerance test (OGTT) was conducted with ingestion of 75 grams glucose solution. Blood samples were collected prior to ingestion (fasting) and 2 h post-prandial. A fasting glucose value of ≥ 5.1 mmol/l and 2 h OGTT value of ≥ 8.5 mmol/l was grouped as GDM, according to criteria set by IADPSG.

To analyze the data, participants were grouped according to GDM status based on OGTT values at second trimester (18). Baseline (1st trimester) anthropometric and biochemical data were utilized to assess the status of full MetS and its five components as present/absent (dichotomous data) according to the criteria set in the National Cholesterol Education Programme Adult Treatment Panel III (NCEP-ATP III) where Full MetS was identified as present when at least three out of five components are present (19).

- Waist circumference of >88 cm.
- Fasting glucose >5.6 mmol/L.
- HDL-Cholesterol <1.30 mmol/L.
- Triglycerides >1.7 mmol/L.
- Systolic blood pressure >130 mmHg and/or diastolic blood pressure >85 mmHg.

The data was analyzed using SPSS (Version 22.0). To assess the normal distribution of the preliminary data, Kolmogorov-Smirnov test was employed. Continuous normally distributed variables were presented as mean ± standard deviation (SD) while median (25th and 75th percentile) was used for continuous non-normal variables. Categorical variables full MetS and its individual components were presented as percentages (%). Independent Student T-test and Mann-Whitney *U*-test were used to test for differences between groups for normal and non-normal variables, respectively. Chi-square test (McNemar 2 × 2 contingency table) was used for categorical variables. Data was presented as correlation coefficient (r) and the associated p-value. Associations of circulating levels of biochemical parameters at 1st trimester with 2nd trimester OGTT levels were done using Pearson and Spearman correlations for normal and non-normal variables, respectively. Logistic regression was performed using GDM status as a dependent variable and full MetS or its individual components as independent variables. Data was presented as odds ratio (95% confidence interval) [OR (95% CI)] and respective p-values representing odds of having different components of MetS at 1st trimester of pregnancy. Different models were employed with model “a” as univariate, and all other models were adjusted accordingly for age (model “b”), BMI (model “c”), and other covariates like parity (model “d”). The statistical analysis was conducted at 95% confidence level and a *p* < 0.05 was considered statistically significant. MS excel 2010 was used to plot figures.

## Results

Fasting glucose and 2 h OGTT values at second trimester were used to diagnose 123 (24.7 %) pregnant women as having GDM according to IADPSG criteria. The baseline (1st trimester) anthropometric and biochemical characteristics of the participants in the two groups were compared for the differences and the results are shown in Table 1. Non-GDM participants were significantly younger and had lower BMI than the GDM group (*p*-values 0.004 and 0.003, respectively. Also, the mean number of children was statistically higher in GDM (*p* = 0.036) compared to non-GDM. Similarly, even in early pregnancy, the levels of fasting glucose, fasting insulin and HbA1c were significantly higher in GDM than non-GDM participants (*p* = 0.013, 0.043, and 0.019, respectively). Interestingly, serum triglycerides at 1st trimester were higher in GDM (1.7 ± 0.8 mmol/l) compared to non-GDM (1.4 ± 0.6) with statistically significant *p*-value of 4.9 × 10^−4^.

**Table 1:** Biochemical characteristics and incidence of MetS components at 1st trimester.

**Parameters**	**GDM**	**Non-GDM**	***P*-values**
Fasting glucose at 2^nd^ trimester (mmol/l)	5.3 ± 1.4	4.5 ± 0.7	
Glucose at 2^nd^ trimester-2 h OGTT (mmol/l)	10.5 ± 2.6	5.9 ± 1.2	
**ANTHROPOMETRICS (1**^**ST**^ **TRIMESTER)**			
Gestational age (weeks)	11.4 ± 2.9	11.4 ± 2.6	0.98
Age (years)	31.2 ± 6.1	28.6 ± 5.3	0.004
Parity #	2 (2.0, 5.5)	2 (1.0, 4.0)	0.036
BMI (kg/m^2^)	31.6 ± 7.1	28.1 ± 6.4	0.003
Waist (cm)	93.1 ± 12.5	90.7 ± 13.5	0.69
Hips (cm)	108.9 ± 10.4	107.9 ± 12.4	0.13
Systolic blood pressure (mmHg)	112.6 ± 13.8	113.2 ± 12.9	0.37
Diastolic blood pressure (mmHg)	68.3 ± 10.2	69.5 ± 45.7	0.76
**BIOCHEMICAL CHARACTERISTICS (1**^**ST**^ **TRIMESTER)**			
Glucose (mmol/l)	5.4 ± 1.3	4.9 ± 1	0.013
Triglycerides (mmol/l)	1.7 ± 0.8	1.4 ± 0.6	4.9 × 10^−4^
Total Cholesterol (mmol/l)	5.4 ± 1.4	5 ± 1.1	0.07
HDL-cholesterol (mmol/l)	1.3 ± 0.4	1.4 ± 0.3	0.14
LDL-cholesterol (mmol/l)	3.1 ± 0.9	3 ± 0.8	0.10
Calcium (mmol/l)	2.2 ± 0.2	2.2 ± 0.2	0.72
Alkaline phosphatase (mmol/l)	10.2 ± 3.7	10.0 ± 4.5	0.42
PTH (pg/ml) #	10.9 (2.3, 12.2)	10.9 (2.1, 13.3)	0.56
**MetS COMPONENTS (1**^**ST**^ **TRIMESTER)**			
Central Obesity (%) $	66.7	59.9	0.25
Hyperglycemia (%) $	30.9	16.5	0.002
Low HDL-C (%) $	55.1	47.1	0.12
Hypertriglyceridemia (%) $	40.7	24.8	0.003
Hypertension (%) $	15.3	10.8	0.16
Full MetS (%) $	25.1	13.4	0.008

*Data presented as Mean ± Standard Deviation for continuous normal variables and medians (25^th^ percentile−75^th^ percentile) for continuous non-normal variables; # indicates non-normal variables; categorical variables ($) are presented as frequency (%). Independent sample t-test and Mann-Whitney U-test are used for significance testing for Gaussian and non-Gaussian variables, respectively, while chi-square test of independence was used for categorical variable(s). P-value < 0.05 is considered as significant*.

For the MetS components at first trimester, the prevalence of hyperglycemia (fasting glucose ≥5.6 mmol/l) was higher in the GDM group than the non-GDM even in early pregnancy (30.9% vs. 16.5%, *p* = 0.002). Interestingly, 40.7% (50/123) in GDM group had hypertriglyceridemia compared to 24.7% (93/375) in non-GDM group (*p* = 0.003). Also, the full MetS was prevalent in 25.1% in the GDM group compared to 13.4% in non-GDM group (*p* = 0.008).

**Figure 1:**
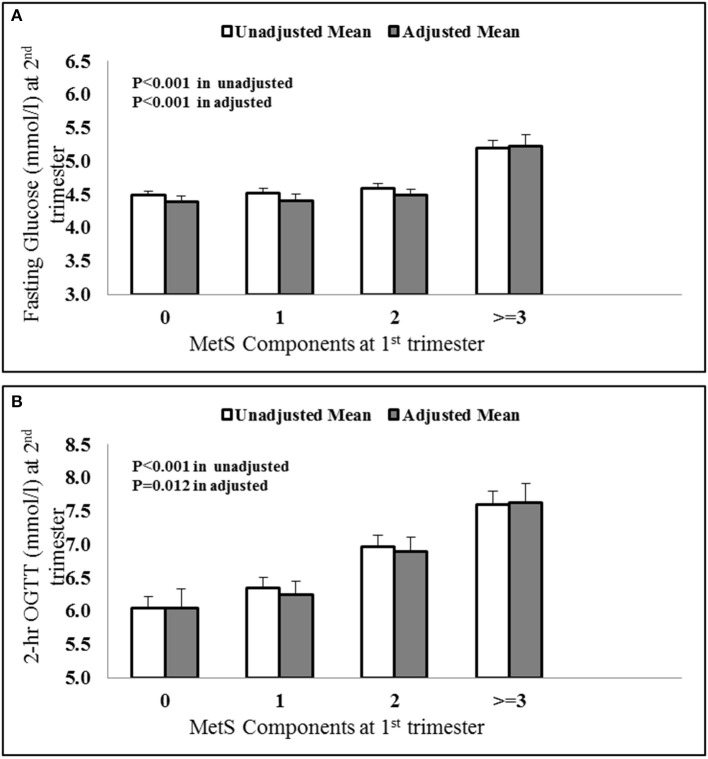
Increasing **(A)** fasting glucose and **(B)** OGTT values at 2^nd^ trimester vs. MetS components at first trimester. The values were adjusted with confounders like age, BMI and parity.

A univariate analysis was done using fasting glucose and OGTT values of 2nd trimester as dependant variables and MetS components as independent variables. Figure 1 shows the average value of fasting glucose (Figure 1A) and OGTT values (Figure 1B) in the later phase of pregnancy for pregnant women with 0, 1, 2, and ≥3 MetS components in their first trimesters. The results show a statistically significant increase in both fasting glucose and OGTT values for higher MetS components.

**Figure 2:**
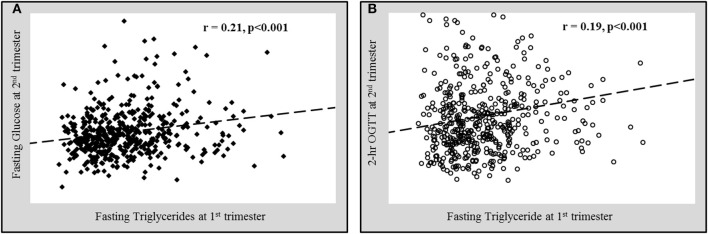
Significant positive correlation of fasting triglycerides at 1^st^ trimester with **(A)** fasting glucose and **(B)** 2 h oral glucose tolerance test values (OGTT) at 2^nd^ trimester.

The associations of various anthropometric and biochemical parameters recorded at the first trimester of pregnancy with fasting glucose and 2 h OGTT levels taken at second trimester were shown in Table 2. As expected, glycemic indexes at 1st trimester (fasting glucose and HbA1c) showed a significant positive correlation with both fasting glucose and 2 h OGTT values at 2nd trimester. In addition to glycemic indexes, age and parity both showed a significant positive correlation with fasting glucose and 2 h OGTT values at 2nd trimester. BMI showed a significant positive correlation while HDL-cholesterol showed a significant inverse correlation with fasting glucose at 2nd trimester. Interestingly, the serum triglycerides at 1st trimester were found to be significantly positively correlated with both fasting glucose (Figure 2A) and 2 h OGTT values (Figure 2B) at later phase of pregnancies (2nd trimester) (*r* = 0.21, *p* < 0.001 and *r* = 0.18, *p* < 0.001 respectively).

**Table 2:** Associations of clinical parameters at 1st trimester with Serum Glucose (fasting and 2h OGTT) at 2nd trimester.

**Parameters**	**2**^****nd****^ **trimester glucose (fasting)**		**2**^****nd****^ **trimester 2h OGTT**	
	***r***	***p*-value**	***r***	***p*-value**
Age	0.14	0.002	0.22	2.1 × 10^−6^
Parity	0.16	0.007	0.13	0.03
BMI	0.16	4.6 × 10^−4^	0.08	0.09
Waist	0.10	0.08	0.07	0.22
Hips	0.15	0.007	0.04	0.52
Systolic BP	−0.07	0.21	−0.003	0.96
Diastolic BP	−0.04	0.48	0.03	0.62
Fasting Glucose	0.24	8.5 × 10^−8^	0.19	1.7 × 10^−5^
Triglycerides	0.21	1.8 × 10^−6^	0.18	3.4 × 10^−5^
Total Cholesterol	0.08	0.07	0.09	0.04
HDL–Cholesterol	−0.11	0.02	−0.08	0.10
Calcium	−0.02	0.71	0.02	0.98
Alkaline Phosphatase	0.11	0.16	−0.02	0.79
PTH #	−0.10	0.10	−0.05	0.36

Data presented as correlation coefficient (r) and the associated p-value. Pearson correlation is used for normal variables and Spearman correlation for non-normal variables (#).

The presence of full MetS and its components at first trimester was checked for their association with GDM risk by binomial regression and the results were shown as odds ratio (OR) and 95% confidence intervals (CI) in Table 3. Although the odds of central obesity, low HDL-cholesterol and hypertension were higher in GDM vs. non-GDM participants, the *p*-values associated with these odds ratio were not significant. The odds of hyperglycemia, even at 1st trimester of pregnancy, was significantly higher in GDM than in non-GDM participants even after multiple adjustments (OR: 2.13, 95% CI: 1.1 to 4.3, *p* = 0.038). Similarly, the odds of hypertriglyceridemia at first trimester was significantly higher in the GDM group than those without (OR: 2.07, 95% CI: 1.3 to 3.4, p: 0.003) even after multiple adjustments with confounders like age, BMI and parity (OR: 1.82, 95% CI: 1.1 to 3.7, *p* = 0.04). The odds of having full MetS at first trimester was significantly higher in GDM than the non-GDM participants, however it lost the statistical significance after adjustments with age, BMI, and parity.

**Table 3:** Odds of full MetS and its components at 1^st^ Trimester among Saudi pregnant women.

**MetS Component**	**OR (95% CI)**	***p*-value**
**CENTRAL OBESITY**		
Model a	1.46 (0.8–2.6)	0.20
Model b	1.14 (0.6–2.1)	0.68
Model c	1.15 (0.6–2.3)	0.69
Model d	1.16 (0.5–2.5)	0.69
**HYPERGLYCEMIA**		
Model a	2.27 (1.3–3.8)	0.002
Model b	1.90 (1.1–3.4)	0.03
Model c	2.05 (1.1–3.7)	0.08
Model d	2.13 (1.1–4.3)	0.038
**LOW HDL-CHOLESTEROL**		
Model a	1.38 (0.8–2.2)	0.20
Model b	1.49 (0.8–2.6)	0.14
Model c	1.53 (0.9–2.6)	0.12
Model d	1.49 (0.8–2.8)	0.22
**HYPERTRIGLYCERIDEMIA**		
Model a	2.07 (1.3–3.4)	0.003
Model b	1.81 (1.1–3.2)	0.03
Model c	1.86 (1.1–3.6)	0.03
Model d	1.82 (1.1–3.7)	0.04
**HYPERTENSION**		
Model a	1.65 (0.7–3.7)	0.23
Model b	1.44 (0.6–3.4)	0.39
Model c	1.54 (0.6–3.7)	0.33
Model d	1.53 (0.6–3.9)	0.34
**FULL MetS**		
Model a	2.16 (1.2–3.8)	0.008
Model b	1.71 (1.1–3.3)	0.04
Model c	1.74 (0.9–3.2)	0.06
Model d	1.73 (0.8–3.6)	0.14

*Data presented as Odds Ratio (95% confidence interval) [OR (95% CI)] and respective p-values of having different components of MetS at 1^st^ trimester of pregnancy in participants with GDM vs. those without GDM among the study population. Model “a” is univariate. All other models are additionally adjusted for age (model “b”), BMI (model “c”) and other covariates like parity (model “d”)*.

**Figure 3:**
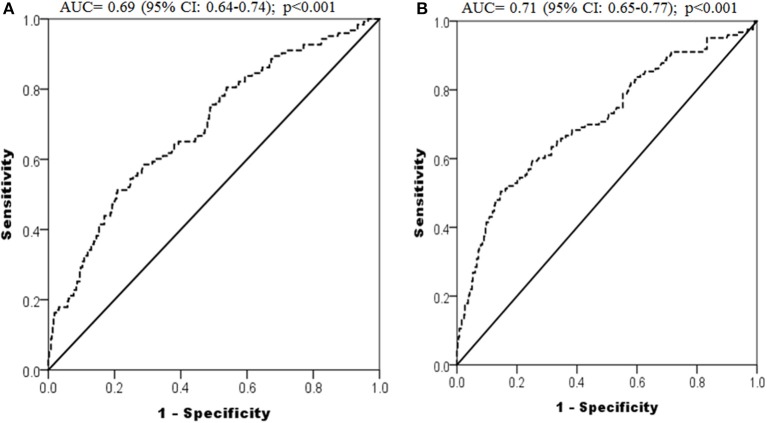
ROC curves depicting predictive power of first trimester fasting glucose **(A)** and triglycerides **(B)** for development of GDM.

The receiver operating characteristic (ROC) analysis was conducted using fasting glucose (Figure 3A) and triglyceride (Figure 3B) levels in the first trimester for predicting GDM and revealed an area under to curve (AUC) of 0.69 (95% CI: 0.64 to 0.74, *p* < 0.001) and 0.71 (95% CI: 0.65 to 0.77, *p* < 0.001) respectively. The cut-off of fasting glucose and triglycerides in the first trimester for predicting GDM, obtained in this ROC analysis, was 5.25 and 1.76 mmol/l respectively, with sensitivities of 51.2 and 50.4% and specificities of 79.2 and 85.4% respectively.

## Discussion

In this study, the presence of MetS components, especially hyperglycemia and hypertriglyceridemia in the first trimester of pregnancy, precede the development of GDM. This effect was independent of established risk factors for GDM like age, BMI and multiparity. These observations highlight the importance of early pregnancy biomarkers that can help identify women at risk of developing GDM early in their pregnancies which in turn provides an opportunity for an early targeted intervention to reduce GDM-related complications.

Early GDM diagnosis and treatment greatly reduces potential complications for mothers and babies (20) and is cost effective in terms of improving outcomes like decreased rates of preeclampsia, cesarean sections, neonatal hypoglycemia, and intensive care unit admissions etc. (21). However, the current screening procedure for GDM in the later stages of pregnancy leaves a limited opportunity for an early intervention (22). An early GDM risk identification such as altered biomarkers that precede hyperglycemia offers several clinical benefits including an early lifestyle intervention targeting gestational weight gain, which potentially reduces GDM and other clinical outcomes in the short and long term (23, 24).

In this study, the prevalence of hyperglycemia in the first trimester of pregnancy was significantly higher in women diagnosed with GDM in later half of the pregnancy than those without (30.9% vs. 16.5%, *p* = 0.002). After adjustment for confounders like age, BMI and multiparity, the odds of first trimester hyperglycemia in GDM vs. non-GDM participants was 2.1 (95% CI 1.1 to 4.3). Our data suggested that the fasting glucose level >5.25 mmol/l in first trimester can predict GDM with sensitivity and specificity of 51.2 and 79.2% respectively. This is in line with several findings which showed a high prevalence of GDM in the first trimester of pregnancy (25, 26). Similarly, Seshiah et al. (27) reported a GDM prevalence of 16.3% GDM diagnosed within first 16 weeks of pregnancy. These findings suggest that hyperglycemia antedates the threshold of GDM normally seen in the later part of the pregnancy. One possible explanation suggests that β-cell dysfunction found in GDM occurs because of a chronic insulin resistance already present before pregnancy (28) or that the fetus acts as an antigenic load which triggers events leading to insulin resistance and inadequate hepatic insulin extraction found in GDM (29). Regardless of the causes, screening for GDM in the later stage of pregnancy results in possible prolonged exposure of fetus to hyperglycemia which is an important issue that can lead to persistent insulinemia and accelerated fetal growth (30).

It is known that pregnancy-related hypertriglyceridemia can cause hormonal changes affecting lipid metabolism. Maternal triglyceride levels, especially in the third trimester were found to be strong predictors of birth weight regardless of GDM status (31, 32). However, it is not clear whether hypertriglyceridemia in the first trimester independently predicts GDM. In this study, hypertriglyceridemia was significantly higher in women with GDM (40.7 vs. 24.8%, *p* = 0.003) and the odds of first trimester hypertriglyceridemia after controlling for multiple confounders was significant [1.82 (95% CI 1.1 to 3.7)]. ROC analysis suggested that the first trimester triglyceride levels of >1.76 mmol/l was able to predict GDM with a sensitivity and specificity of 50.4 and 85.4%, respectively. These findings are consistent with a recent study by Shen et al. (33) which demonstrated that the highest quartile of triglyceride levels was associated with increased risk of GDM [OR: 2.04 (95% CI 1.41 to 2.95). Similarly, Brisson et al. (34) demonstrated that the hypertriglyceridemic waist phenotype, characterized by abdominal obesity coupled with hypertriglyceridemia, in the first trimester may be useful in early screening for GDM. These findings are of clinical importance since elevated maternal triglycerides at first trimester could be reduced through dietary modifications and hence decrease GDM risk.

MetS and GDM are two important metabolic disorders that are increasing worldwide and Saudi Arabia is no different. In our study, the prevalence of full MetS was significantly higher in GDM than non-GDM participants (25.1 vs. 13.4%, *p* = 0.008. Even though the odds of having full MetS at first trimester in GDM compared to non-GDM participants was not significant after controlling for multiple confounders (e.g., age, BMI, and multi-parity), the univariate analysis showed that the mean OGTT values at second trimester increased parallel to the number of MetS components. These findings could be explained with overlapping pathogenic pathways such as insulin resistance, hyperlipidemia and impaired endothelial function in MetS and GDM. Also, there are studies that link central obesity, a component of MetS, with GDM risk (35) and with higher risk of perinatal complications (36). Whatever may be the pathophysiology behind the development of GDM, diagnosing it or identifying pregnant women at risk in early pregnancy is important in order to initiate early lifestyle changes that may affect the course of the disease and assessing MetS components in the early pregnancy may be used specially because it is easy to incorporate this assessment in the routine pregnancy tests.

The strengths of this study include (a) the cohort design where data on MetS components was collected prospectively to establish link with GDM incidence; (b) binomial regression analysis was adjusted with known confounders of GDM like age; BMI and multiparity; (c) homogeneity of the study group; (d) the study population have a high prevalence of both MetS (37) and GDM (38), thus presenting perfect samples to study such diseases; and (e) ROC analysis done in this study provides cut-offs of early pregnancy hypertriglyceridemia and hyperglycemia predicting high risk of GDM.

The authors acknowledge certain limitations of this study. First, although the data was adjusted for confounders like age, BMI, and multi-parity, other known factors to influence GDM risk like pre-pregnancy BMI, dietary habits, level of physical activity, and education status were unavailable and as such were not included in the analysis. The findings are limited to the Arabian cohort and further studies on other ethnically homogenous populations should be conducted. Also, large randomized controlled intervention trials testing the effect of early lifestyle changes targeting different components of MetS for the prevention of GDM and its complications should be planned. Lastly, while the sample size is considerably small, it is nevertheless robust in power and considered adequate.

In conclusion, our data suggests that the incidence of GDM among Saudi pregnant women is strongly associated with early manifestations of all MetS components, especially hyperglycemia and hypertriglyceridemia. These findings merit clinical importance since early assessment of MetS in pregnancy may identify those at greatest risk for GDM who will benefit the most from pregnancy-friendly lifestyle changes (dietary modifications and daily physical activity). However, elucidation of the complex processes underlying these findings requires further study.

## Data Availability Statement

The datasets generated for this study are available on request to the corresponding author.

## Ethics Statement

The studies involving human participants were reviewed and approved by Ethics Committee of the College of Science, King Saud University (Approval#14/4067/IRB, date: 11.02.2014). The patients/participants provided their written informed consent to participate in this study.

## Author Contributions

NA-D and MF designed the study. SA-M, IT, AA-A, and MA worked in the methodology. KW, AA, and NA did the formal analysis. SS and KW helped in the data curation. The original draft was written by KW. Manuscript review and editing process was handled by SA-M, MF, SS, and KW. The study supervision was done by NA-D.

### Conflict of Interest

The authors declare that the research was conducted in the absence of any commercial or financial relationships that could be construed as a potential conflict of interest.
